# Tislelizumab plus chemotherapy versus chemotherapy as first-line treatment for extensive-stage small cell lung cancer: A cost-effectiveness analysis

**DOI:** 10.1371/journal.pone.0320189

**Published:** 2025-03-25

**Authors:** Zhiwei Zheng, Huide Zhu, Ling Fang

**Affiliations:** Department of Pharmacy, Cancer Hospital of Shantou University Medical College, Shantou, China; Inha University Hospital, KOREA, REPUBLIC OF

## Abstract

**Objective:**

This study aims to conduct a cost-effectiveness analysis of tislelizumab in combination with platinum and etoposide compared to the standard treatment of etoposide and platinum as first-line therapy for extensive-stage small cell lung cancer(ES-SCLC) from the Chinese medical system perspective.

**Methods:**

A partitioned survival model was developed utilizing data from the RATIONALE-312 trial to accurately simulate the clinical and economic outcomes of both treatment arms. This model incorporates three distinct health states, namely progression-free survival, disease progression, and death. These states are exclusive of each other, and patients can transition between them as their disease progresses.The model accounted for various cost components such as drug therapy, management of adverse events, disease progression, and overall survival. To evaluate the cost-effectiveness of the interventions, quality-adjusted life-year (QALY) and incremental cost-effectiveness ratio (ICER) were chosen as the metrics. The analysis employed a willingness to pay (WTP) threshold of $39,855.79 per QALY. Additionally, sensitivity analyses were conducted to assess the robustness and reliability of the model.

**Results:**

The tislelizumab group had a total cost of $52,749.69, whereas the chemotherapy group’s total expenses amounted to $8,811.62. Additionally, the tislelizumab group experienced a gain of 2.21 QALY compared to the chemotherapy group, albeit incurring an additional cost of $43,938.07. Consequently, this led to an ICER of $19,881.48, which falls below the Chinese WTP threshold of $39,855.79. Sensitivity analyses confirmed the robustness of the findings across a range of scenarios.

**Conclusion:**

This cost-effectiveness analysis based on the RATIONALE-312 trial demonstrates that tislelizumab plus platinum and etoposide is a cost-effective treatment option for ES-SCLC compared to the standard chemotherapy from the Chinese medical system perspective.

## 1. Introduction

Lung cancer represents a significant global health burden and is responsible for a substantial number of deaths worldwide. It is estimated that approximately 18.7 percent of annual deaths are attributed to lung cancer [1]. Small cell lung cancer (SCLC) is a highly lethal form of lung cancer, characterized by its aggressive nature and neuroendocrine origins [2].It accounts for approximately 15% of all diagnosed lung cancer cases, making it a significant contributor to the burden of this disease [3]. Unfortunately, one noteworthy aspect of SCLC is that a significant majority of patients, surpassing 60%, receive their diagnosis when the cancer has already metastasized to distant sites [4]. This delayed diagnosis greatly reduces the chances of successful treatment and contributes to the poor prognosis associated with this disease. In fact, the overall 5-year survival rate for extensive stage small cell lung cancer(ES-SCLC) remains dismally below 5% [5].Treatment options for SCLC primarily include chemotherapy and radiation therapy, as surgery is generally not feasible due to the advanced stage of the disease at diagnosis[6]. Platinum-based chemotherapy regimens, often combined with etoposide, have shown efficacy in achieving tumor response and improving patient outcomes [7,8]. However, despite initial response rates, SCLC frequently relapses and acquires resistance to therapy [9]. The development of immunotherapies therapies holds promise for improving the management of SCLC [10].The use of immune checkpoint inhibitors (ICIs), specifically programmed death-1 (PD-1) and programmed death-ligand 1 (PD-L1), has revolutionized the treatment approach for numerous solid tumors. These inhibitors present a promising therapeutic strategy for addressing the treatment complexities associated with ES-SCLC[11].Preliminary clinical trials and studies have demonstrated encouraging results with the incorporation of ICIs in the management of ES-SCLC. Significant clinical benefit of combining ICIs with chemotherapy in the first-line setting for ES-SCLC has been demonstrated in key trials, including the IMpower133 and CASPIAN trials [12,13]. These studies have shown improved overall survival and progression-free survival when ICIs, such as atezolizumab and durvalumab, are added to platinum-based chemotherapy regimens.Furthermore, the success of ICIs in ES-SCLC has prompted investigations into their use in the second-line and maintenance settings. Preliminary data suggests durable responses and improved survival when PD-1/PD-L1 inhibitors, such as pembrolizumab or nivolumab, are administered as monotherapy following first-line chemotherapy [14,15]. These findings highlight the potential of ICIs to fundamentally alter the treatment paradigm for ES-SCLC by providing durable responses and improving overall outcomes.

Recently, in a phase 3 RATIONALE-312 study conducted on a cohort of patients with ES-SCLC, tislelizumab in combination with platinum and etoposide as a first-line treatment demonstrated promising results. The overall response rate (ORR) was found to be 68%, indicating a significant therapeutic effect. Furthermore, the median progression-free survival (PFS) was determined to be 4.7 months, and the median overall survival (OS) was observed to be 15.5 months [16]. The results of the study provide compelling evidence for the therapeutic potential of tislelizumab in combination with chemotherapy for the treatment of patients with extensive-stage small cell lung cancer.The results demonstrate that this treatment approach not only leads to tumor responses but also improves overall outcomes for these patients. Based on this clinical trial data, on June 28, 2024, China National Medical Products Administration (NMPA) approved tirelizumab in combination with etoposide and platinum-based chemotherapy regimen for first-line treatment of ES-SCLC, which has now become the recommended treatment method in China medical institution guidelines.

While tislelizumab in combination with chemotherapy has demonstrated the ability to prolong the survival duration of patients afflicted with ES-SCLC, it is important to recognize that this therapeutic approach comes with a financial burden compared to the use of chemotherapy alone. With the introduction of tislelizumab treated in ES-SCLC patients, it is crucial to determine whether the increased expenses associated with this novel drug can be justified by the potential health benefits within the limitations of the healthcare budget. Limited evidence is currently available regarding the cost-effectiveness of tislelizumab in ES-SCLC. Hence, the objective of this research is to evaluate the cost-effectiveness of integrating tislelizumab into the treatment of ES-SCLC patients from the perspective of the Chinese healthcare system.

## 2. Methods

### 2.1. Model establish and assumptions

Based on the clinical data from the RATIONALE-312 trial, we have developed a partitioned survival model to assess the cost-effectiveness of various treatment options for metastatic cervical cancer. This model comprises three distinct health states which are mutually exclusive: progression-free survival, disease progression, and death. Patients can transition between these states as their condition evolves over time.

**Fig 1:**
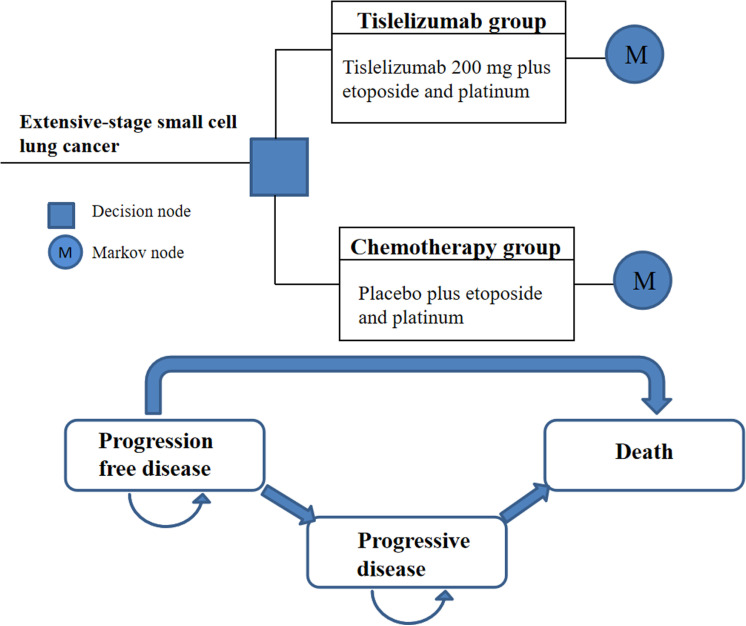
The Markov model.

The study primarily focused on patients diagnosed with ES-SCLC. The model participants were randomly assigned to two treatment groups: one group receiving a combination of tislelizumab and chemotherapy, and the other group receiving chemotherapy alone. To simulate the progression of the disease, a markov model was created. The model included three distinct health states: progression-free disease, progressive disease, and death. These states were assumed to be mutually exclusive, meaning that each patient could only be in one state at a time. It was initially assumed that all patients started in the progression-free disease state and could either remain in that state or transition to another state in each cycle (Fig 1).

The total duration of the simulation in this model was set to 10 years, which was determined based on the overall 5-year survival rate of patients with ES-SCLC being less than 5%.The clinical data utilized in this study were derived from the phase III RATIONALE-312 trial. The RATIONALE-312 trial administered tislelizumab or chemotherapy every 3 weeks, thus the treatment period in the model was also set at 3 weeks. To ensure the accuracy of the model, the simulated survival curves were internally validated against the survival curves observed in the RATIONALE-312 trial. Additionally, the PFS and OS curves generated by the simulation were externally validated against those observed in the ASTRUM-005 clinical trial(Supplementary Figure 1) [17]. Importantly, the simulation results closely aligned with the actual trial outcomes, providing further evidence of the reliability and validity of the model.

We established the willingness-to-pay (WTP) threshold at US$39,855.79 per quality-adjusted life year (QALY) [18]. This threshold corresponds to three times the gross domestic product (GDP) per capita for the year 2023 [19]. All costs related to this study were converted from Chinese renminbi (RMB) to United States dollars (USD) using the average official exchange rate for the preceding year, 2023, at a rate of 1 USD equivalent to 7.05 RMB [19].Statistical analysis and modeling for this study were performed using TreeAge Pro 2022.

### 2.2. Model input treatment

Our model assumes a study population consistent with the RATIONALE-312 study population. The clinical data for the model treatment was obtained from the phase III RATIONALE-312 trial. The treatment regimen consisted of four 21-day cycles of intravenous administration of either tislelizumab at a dose of 200 mg or placebo every 3 weeks. This was given in combination with etoposide, which was administered intravenously at a dose of 100 mg/m^2^ over the course of days 1 to 3 of each 21-day cycle. In addition, a platinum agent, either cisplatin at a dose of 75 mg/m^2^ or carboplatin(area under the plasma concentration-time curve = 5) on day 1 of each 21-day cycle. Following the completion of the initial treatment, a maintenance phase was initiated, wherein patients were administered either intravenous tislelizumab (200 mg) or a placebo every 3 weeks.

The treatment duration utilized in our model was based on the RATIONALE-312 clinical trial. In particular, the median duration of treatment in the tislelizumab group was found to be 19.4 weeks (15.4-41.1), while in the chemotherapy group, it was 19.1 months (17.3-25.7).

The study model was designed to incorporate treatment-related adverse events, which were accounted for based on the occurrence of grade 3-4 serious adverse events (SAEs) that resulted from the treatment intervention. Specifically, adverse events with an incidence exceeding 1% in the RATIONALE-312 clinical trial were included as integral components of the model.

A total of 306 patients (67%) in the intent-to-treat population underwent subsequent systemic anticancer therapy. Specifically, this included 136 patients (60%) from the tislelizumab group and 170 patients (74%) from the chemotherapy group. The most commonly utilized subsequent systemic anticancer therapy in both treatment groups was conventional chemotherapy. In the tislelizumab group, 124 patients (55%) received this therapy, while in the chemotherapy group, 155 patients (67%) underwent conventional chemotherapy.

To conduct a comprehensive cost-effectiveness analysis, we assumed the irinotecan plus cisplatin chemotherapy regimen as the second-line treatment option for both patient groups. However, it should be noted that the optimal choice for third-line therapy following the failure of second-line therapy remains uncertain. Consequently, we considered the best supportive treatment to be the most effective option when the disease progressed once again in our study model. There was uncertainty regarding the selection of platinum-based drugs, including cisplatin and carboplatin. Our model operates under the assumption that patients had an equiprobable chance of receiving either of these medications. To evaluate their influence on economic outcomes, a sensitivity analysis will be conducted on these variables.

### 2.3. Transfer probability data

Based on the Kaplan-Meier survival curves obtained from the RATIONALE-312 clinical trial, we employed the get data graph digitizer 2.25 software to extract data points and re-plot the model survival curves. Subsequently, we utilized the R software to calculate the model transfer probability data. In order to estimate the distributions of survival probabilities, various distributions such as gamma, log-log, log-normal, gompertz, exponential, and weibull were evaluated. The selection of the most appropriate distribution was based on a combination of visual inspection, data measurement such as akaike’s information criterion (AIC) and bayesian information criterion (BIC) minimum evaluations. The outcomes are depicted in S2 fig and S1 Table.

The log-logistic distribution was subsequently chosen as the optimal model for simulating the distribution of the PFS and OS curves for the two treatment schemes. To ensure the effectiveness and broaden the applicability of our model beyond the duration of the clinical trial follow-up, we employed a simulation approach to generate survival times based on the log-logistic distribution.To calculate the survival function of the log-logistic distribution over time, we utilized the formula S(t) =  1/(1 +  λt^γ^) [20]. The estimated values of these parameters can be found in Table 1.

**Table 1:** Log-logistic survival Estimates parameters.

Variable	shape (γ)	scale (λ)
OS survival curve		
Tislelizumab group	1.78	0.0068
Chemotherapy group	2.37	0.0019
PFS survival curve		
Tislelizumab group	1.92	0.035
Chemotherapy group	3.97	0.0022

### 2.4. Cost and utility input

From a Chinese healthcare system perspective, the study primarily focuses on the collection of data related to direct medical costs. These costs include the costs of drugs, the management of adverse events, the subsequent treatment, the best supportive care, follow-up, and the medical examinations. Chemotherapy doses are prescribed using a standardized model that relies on a number of key factors. These factors include an assumed body weight of 60 kg, a creatinine clearance rate of 70μmol/L, and a body surface area of 1.72 m^2^.

We obtained drug cost data from the drug database (https://data.yaozh.com/). This database provides information on winning bid prices of drugs from various regions in the country. In order to accurately represent the cost data, we calculated the median value of the winning bid prices and incorporated these values into our model.

In this study, health utility values were employed to evaluate the associated quality of life. The scale used ranged from 0 to signify the lowest quality of life to 1 indicating the highest quality of life, with all utility values spanning between 0 and 1. Nevertheless, the RATIONALE-312 clinical trial did not explicitly provide data on these utility values. Consequently, we sourced these values from existing literature to bolster the rigor and academic rigor of our analysis. Additionally, our model also incorporated utility values for SAEs. Table 2 presents the input information about the cost and utility values.

**Table 2:** The input parameters to the model.

Variable	Baseline value	Range		Distribution	Source
		Minimum	Maximum		
Tislelizumab group SAEs (grade ≥ 3) incidence					
Anaemia	0.16	–	–	Beta	[16]
Leukopenia	0.11	–	–	Beta	[16]
Neutropenia	0.56	–	–	Beta	[16]
Thrombocytopenia	0.19	–	–	Beta	[16]
Chemotherapy group SAEs (grade ≥ 3) incidence					
Anaemia	0.17	–	–	Beta	[16]
Leukopenia	0.10	–	–	Beta	[16]
Neutropenia	0.55	–	–	Beta	[16]
Thrombocytopenia	0.25	–	–	Beta	[16]
Drug cost (US dollar $)					
Tislelizumab per mg	1.78	1.34	2.23	Gamma	[21]
Etoposide per mg	0.45	0.34	0.56	Gamma	[21]
Cisplatin per mg	0.22	0.17	0.28	Gamma	[21]
Carboplatin per mg	0.086	0.06	0.11	Gamma	[21]
Costs of SAEs (grade ≥ 3) events per cycle ($)					
Anaemia	531.72	398.79	664.65	Gamma	[22]
Leukopenia	461.25	345.94	576.56	Gamma	[22]
Neutropenia	84.21	63.16	105.26	Gamma	[23]
Thrombocytopenia	1054.00	790.50	1317.50	Gamma	[23]
Other					
Subsequent treatment	854.05	640.54	1067.56	Gamma	[23]
Best supportive care per cycle	359.52	269.64	449.40	Gamma	[23]
Follow-up cost per cycle	55.60	41.70	69.50	Gamma	[23]
Routine laboratory examinations per cycle	92.50	69.38	115.63	Gamma	[24]
Abdominal CT per cycle	105.90	79.43	132.38	Gamma	[24]
Utility value					
Progression-free disease	0.67	0.50	0.84	Beta	[25]
Progressive disease	0.47	0.35	0.59	Beta	[25]
Anaemia	0.073	0.05	0.09	Beta	[22]
Neutropenia	0.2	0.15	0.25	Beta	[22]
Thrombocytopenia	0.19	0.14	0.24	Beta	[23]
Leukopenia	0.20	0.15	0.25	Beta	[23]
Body surface area(m^2^)	1.72	1.29	2.15	Beta	[26]
Discount rate	0.05	0.04	0.06	Beta	[ 18 ]

### 2.5. Sensitivity analysis

In this study, we performed rigorous stability analyses on our model, including one-way sensitivity analyses and probabilistic sensitivity analyses. In the one-way sensitivity analysis, we systematically varied the input parameters by ± 25% of the base value to evaluate the potential impact on the incremental cost-effectiveness ratio (ICER). The results of the one-way sensitivity analysis were visually represented using a tornado diagram.

For the probabilistic sensitivity analysis(PSA), we adopted the Monte Carlo simulation technique with 10,000 iterations. In this technique, the input parameters were adjusted according to predefined distribution functions. Specifically, costs were assumed to follow a gamma distribution, while utilities and platinum drug proportion parameter were modeled using a beta distribution. By incorporating the uncertainty associated with these parameters, the PSA allowed us to more comprehensively assess the overall uncertainty and variability of the model results. The results of the probabilistic sensitivity analysis were presented in the form of a scatter plot.

### 2.6. Ethics approval and consent to participate

The cost-effectiveness analysis conducted in this study involved the extraction of data from previously published clinical trials. As the study did not involve any research on human subjects, it was deemed exempt from ethical review by the Hospital Research Ethics Committee, which granted a waiver for ethical approval.

## 3. Results

### 3.1. Base result

The tislelizumab group had a total cost of $52,749.69, while the chemotherapy group’s total costs amounted to $8,811.62. The tislelizumab group gained an incremental 2.21 QALYs but incurred an additional cost of $43,938.07 compared to the chemotherapy group.

To evaluate the cost-effectiveness, the ICER was calculated, which compared the additional cost of the tislelizumab group to the incremental gain in QALYs. The ICER was found to be $19,881.48, which is below the WTP threshold of $39,855.79 in China. This indicates that the tislelizumab regimen may be considered cost-effective within the Chinese healthcare system. Table 3 presents the detailed outcomes obtained from this analysis.

**Table 3:** The results of base case.

Group	Cost ($)	QALYs	Incremental cost ($)	Incremental QALY	ICER ($/QALY)
Tislelizumab group	52,749.69	3.49	43,938.07	2.21	19,881.48
Chemotherapy group	8,811.62	0.28	—	—	—

### 3.2. Sensitivity analysis result

**Fig 2:**
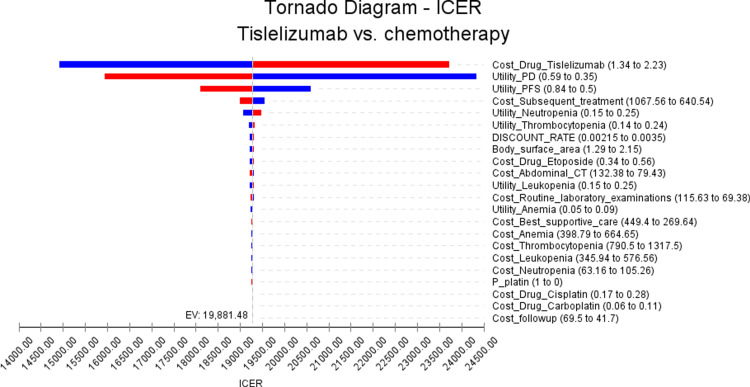
The result of the one-way sensitivity analysis(The cost values in the chart are all in US dollars).

The outcomes of the one-way sensitivity analysis are illustrated in Fig 2. This analysis has found several crucial factors that have had a impact on the ICER. Notably, the cost of tislelizumab, the utility value of progressive disease and progression-free disease have been found to exert a substantial influence on the ICER. It is important to highlight that the cost of drug tislelizumab plays a crucial role in determining the cost-effectiveness of the intervention. A higher drug cost directly affects the ICER, making the treatment less economically viable. However,The sensitivity analysis showed that even when adjusting the cost of the drug, the ICER remained below the WTP threshold.Similarly, the utility values assigned to progressive disease and progression-free disease also play a significant role in shaping the ICER. Utility values reflect the preference or quality of life associated with different health states, and assigning lower values to progressive disease or lower values to progression-free disease may result in higher ICERs.Importantly, even when all uncertain parameters were adjusted, the resulting ICER value did not exceed the threshold of the WTP value. It is essential to emphasize that when these parameters were modified within a margin of ± 25%, no significant alterations to the analysis findings were observed. The findings are in alignment with the conclusion derived from the base-case analysis.

**Fig 3:**
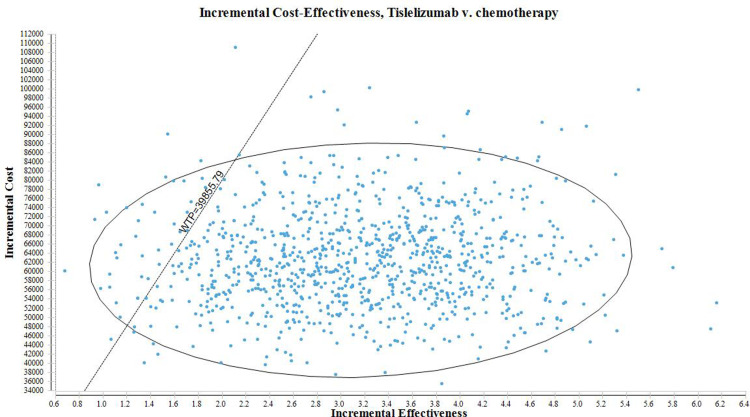
The scatter plot of probabilistic sensitivity analysis(TheWTP value in the chart was in US dollars).

The results of the probabilistic sensitivity analysis are depicted in Fig 3.The plot displays various iterations of the analysis, where each point represents a specific iteration. The vertical axis shows the difference in cost between the interventions being compared, while the horizontal axis represents the difference in health outcomes achieved. The clustering of points in different regions of the plot provides valuable insights into the likelihood of achieving different cost-effectiveness outcomes.Interventions that fall below the linear WTP (willingness-to-pay) threshold are classified as cost-effective. This indicates that these interventions have more favorable cost-effectiveness ratios compared to interventions in other quadrants. The positioning of interventions on the plot allows us to determine their cost-effectiveness status by taking into account both their cost difference and health outcome difference.

By visually analyzing the plot, we can identify clusters of points that fall below the WTP threshold, indicating a higher likelihood of achieving cost-effective outcomes. Based on the findings derived from the analysis, interventions falling below the linear WTP threshold are deemed to be cost-effective. This categorization implies that interventions in this quadrant exhibit a more favorable ICER ratio, indicating lower costs or higher effectiveness compared to interventions in other quadrants. Importantly, when considering the WTP threshold of $39,855.79 per QALY, there is a 96.10% probability of classifying the tislelizumab regimen as a more cost-effective option when compared to the chemotherapy group.

## 4. Discussion

In recent years, the treatment landscape for ES-SCLC has witnessed a significant paradigm shift with the integration of immunotherapy in combination with chemotherapy as the standard first-line therapeutic approach[27]. Combining immunotherapy, particularly immune checkpoint inhibitors, with chemotherapy has shown remarkable efficacy in improving overall survival and progression-free survival rates in ES-SCLC patients. Immune checkpoint inhibitors, such as PD-1 and PD-L1 inhibitors, unleash the suppressed immune response against cancer cells, thereby enhancing the body’s ability to recognize and destroy these malignant cells. Notably, The phase III RATIONALE-312 trials have yielded promising results in assessing the efficacy of Tislelizumab, a novel therapy, when combined with chemotherapy as a front-line treatment for patients diagnosed with ES-SCLC. This combination therapy has demonstrated notable effectiveness in combating the disease, prompting further investigation into its potential benefits. However, it is essential to acknowledge that incorporating tislelizumab into the treatment regimen may have financial implications, potentially resulting in an increase in overall treatment costs. Consequently, it is imperative to conduct a comprehensive assessment of the pharmacoeconomic aspects associated with the integration of tislelizumab therapy. This thorough evaluation aims to determine whether the potential advantages in terms of treatment efficacy outweigh the financial burden, providing a more comprehensive understanding of the therapy’s viability in a real-world, cost-sensitive setting.

Our study findings indicate that the addition of tislelizumab to chemotherapy regimens resulted in a modest but notable increase of 2.21 QALY compared to the group receiving chemotherapy alone. However, it is important to note that this improvement in patient outcomes was accompanied by a substantial incremental cost of $43,938.07.The calculated ICER of $19,881.48, which compares the additional cost of tislelizumab to the associated increase in QALY, was found to be below the WTP threshold commonly accepted in China. This suggests that tislelizumab represents a cost-effective treatment option when considering the specific context of the Chinese healthcare system. To ensure the robustness of our results, a sensitivity analysis was performed, evaluating the potential impact of various variables on the model output. However, the analysis revealed that none of the variables included significantly affected the overall findings, lending further support to the reliability of our conclusions. In light of the cost-effective WTP threshold established at $39,855.79 per QALY in China, our analysis demonstrates that tislelizumab offers considerable cost-effective advantages over chemotherapy alone. Therefore, based on our comprehensive evaluation, we assert that tislelizumab should be considered as a viable treatment option for patients, taking into account both its clinical benefits and economic value.

By considering the cost per QALY and its association with GDP per capita, we can better evaluate the value and feasibility of healthcare interventions[28]. The relationship between the cost per QALY and the GDP per capita of each country is a crucial factor in determining the cost-effectiveness threshold. At this threshold point, the cost per QALY acquired becomes the decisive factor in assessing the value and feasibility of an intervention or policy decision within a healthcare system[29]. The concept of cost per QALY captures the idea that individuals are willing to allocate a specific monetary value to gain an additional year of perfect health or experience a significant improvement in their quality of life[30]. By quantifying this willingness to pay, policymakers and healthcare decision-makers can gain a deeper understanding of the relative value of various healthcare interventions and allocate limited resources in an integrated manner.In our study, we utilized a threshold for the WTP criterion, which was set at three times the GDP, equivalent to $39,855.79 per QALY. This threshold aligns with the guidelines provided by the Chinese Pharmacoeconomics Guidelines 2020^18^. When comparing the cost-effectiveness of tislelizumab to chemotherapy, the ICER was estimated to be $19,881.48 per QALY, which is considerably lower than the WTP threshold of $39,855.79 per QALY. This finding indicates that using tislelizumab as a first-line treatment for ES-SCLC has the potential to be considered cost-effective. Additionally, it is worth noting that although some researchers and scholars have proposed reducing the WTP threshold to 1.5 times the GDP in the current context[31], our data still suggests that the use of tislelizumab for treating ES-SCLC in China remains cost-effective.

We strongly discourage relying solely on cost-effectiveness analyses, including the findings in this study, as the sole basis for making clinical decisions about the availability of tislelizumab. Instead, we suggest using these analyses to inform policy decisions, while continuously improving the economic viability of tislelizumab through cost containment and efficacy enhancement. The escalating cost of medicines has become a significant concern for governments, healthcare systems, and patients worldwide. Not only does the high price of drugs burden healthcare budgets, but it also hinders access to life-saving treatments for patients in need. Cost-effectiveness analyses have emerged as crucial tools for decision-making in healthcare systems, particularly regarding the pricing and reimbursement of medicines[32]. In response to the financial pressures associated with high drug prices, China has implemented a centralized national drug procurement system as a proactive approach. This strategy allows the Chinese government to engage in extensive negotiations with pharmaceutical companies on a large scale, leveraging the country’s considerable purchasing power to secure more favorable prices for important drugs[33]. Undoubtedly, this approach has yielded significant outcomes, as successful negotiations between the Chinese government and pharmaceutical companies have resulted in a reduction in the price of gefitinib by over 50% [34]. Furthermore, there is potential to explore more efficient pharmacological markers to predict the prognosis of tislelizumab for ES-SCLC and accurately target the population that will benefit the most[35]. This enhancement in clinical benefits will significantly contribute to the ongoing improvement of the cost-benefit advantage of tislelizumab for ES-SCLC.

At present, there is a dearth of published studies that investigate the cost-effectiveness of tislelizumab for the treatment of ES-SCLC specifically. Nevertheless, a number of studies have conducted cost-effectiveness analyses on other PD-1 inhibitors in the treatment of ES-SCLC.One study by Zhu et al.evaluated the cost-effectiveness of serplulimab combination therapy in patients with ES-SCLC who had progressed after standard platinum-based chemotherapy[36]. The study utilized a Markov model to simulate disease progression and treatment outcomes. The results showed that the ICER was $12,077 per QALY gained, which had a 91.6% probability of being cost-effective at a WTP of 3 times capita gross domestic product of China in 2021.Another study by Liu et al. examined the cost-effectiveness of pembrolizumab as a first-line treatment for ES-SCLC[37]. The study utilized a Markov model and found that pembrolizumab was associated with higher costs and QALYs compared to standard care. The ICER was estimated at $334,373 per QALY gained. The first-line treatment for ES-SCLC with pembrolizumab plus EP was not cost-effective compared with placebo plus EP From the US payer perspective.

There are some limitations in our study. Firstly, the Phase III clinical trial data used in our study serve as the foundation for our model’s clinical data, assuming that our simulated populations and treatment align with those used in Phase III clinical trials. It is important to note that Phase III clinical trials typically have strict enrollment criteria, resulting in a study population that may not fully represent real-world patients. Therefore, our pharmacoeconomic analysis is based on this limited population, which may impose certain limitations on the generalizability of our findings.Secondly,although we utilized data reconstructions from clinical trials for our cost-effectiveness analyses, it is important to continuously monitor and update these findings as new evidence emerges and as costs and efficacy continue to evolve in this field. Thirdly, our study did not conduct further research on the cost-effectiveness based on different patient subgroup populations. This hinders the ability to ascertain if the results hold true across various patient groups. Fourthly,our study assumed the cost of second-line treatment after disease progression. However, in reality, the choice of subsequent treatment regimen will vary depending on each individual patient. Despite this limitation, the results of one-way sensitivity analyses consistently indicated that the ICER values remained below the threshold of the WTP even when the estimated range of subsequent treatment options was altered. Lastly, our study did not include grade 1 or 2 adverse events in the analysis. While this approach allowed for a more concise model, it may not fully capture the overall impact of treatment-related toxicity on patient prognosis. However, the results of the one-way sensitivity analyses suggested that even if there was a change in the cost of grade 3 or higher adverse events, the ICER values remained below the threshold of willingness-to-pay and did not alter our conclusions.

## 5. Conclusion

Based on the results of this comprehensive study, the utilization of tislelizumab in combination with platinum and etoposide showcases as a promising and cost-effective first-line therapeutic approach for ES-SCLC.

## Supporting information

S1 Table Comparison of survival models distribution. (DOCX)

S1 Fig A: Modes simulation visual overall survival curve ;B: Overall survival curve from ASTRUM-005 clinical trial;C: Modes simulation visual progression-free survival curve;D:Progression-free survival curve from ASTRUM-005 clinical trial.
(TIF)

S2 Fig A: Modes simulation visual progression-free survival curve of tislelizumab group; B:Modes simulation visual progression-free survival curve of chemotherapy group;C:Modes simulation visual overall survival curve of tislelizumab group;D: Modes simulation visual overall survival curve of chemotherapy group.
(JPG)

S1 File Supplementary Survival curve data. (XLSX)
