# Supplementary material for: Tislelizumab plus chemotherapy versus chemotherapy as first-line treatment for extensive-stage small cell lung cancer: A cost-effectiveness analysis
Source: PLoS One. 2025 Mar 25;20(3):e0320189. doi: 10.1371/journal.pone.0320189 (PMC11936185; doi:10.1371/journal.pone.0320189)
Supplement: S1 Table — (DOCX) [file pone.0320189.s001.docx]

**Supplementary Table 1. Comparison of survival models distribution**

|  | AIC | | BIC | |
| --- | --- | --- | --- | --- |
|  | Tislelizumab group | Chemotherap**y** group | Tislelizumab group | Chemotherap**y** group |
| PFS |  |  |  |  |
| Weibull | 1177.738 | 1071.141 | 1184.588 | 1077.917 |
| **Log-logistic** | **1094.946** | **929.890** | **1101.796** | **936.766** |
| Log-normal | 1105.883 | 972.150 | 1112.733 | 979.026 |
| Gompertz | 1150.632 | 1141.807 | 1157.482 | 1148.683 |
| Exponential | 1175.742 | 1144.687 | 1179.167 | 1148.125 |
| Gamma | 1173.572 | 1014.214 | 1180.422 | 1021.090 |
| OS |  |  |  |  |
| Weibull | 1343.318 | 1424.723 | 1350.168 | 1431.599 |
| **Log-logistic** | **1329.627** | **1403.568** | **1336.477** | **1408.240** |
| Log-normal | 1335.057 | 1401.363 | 1341.907 | 1410.444 |
| Gompertz | 1354.717 | 1454.307 | 1361.567 | 1461.183 |
| Exponential | 1354.809 | 1475.468 | 1358.234 | 1478.906 |
| Gamma | 1339.211 | 1413.101 | 1346.061 | 1419.977 |

AIC: Akaike information criterion; BIC: Bayesian Information Criterion; OS: Overall survival; PFS: Progression-free survival;
