# Supplementary figures and images for: Tislelizumab plus chemotherapy versus chemotherapy as first-line treatment for extensive-stage small cell lung cancer: A cost-effectiveness analysis
Source: PLoS One. 2025 Mar 25;20(3):e0320189. doi: 10.1371/journal.pone.0320189 (PMC11936185; doi:10.1371/journal.pone.0320189)

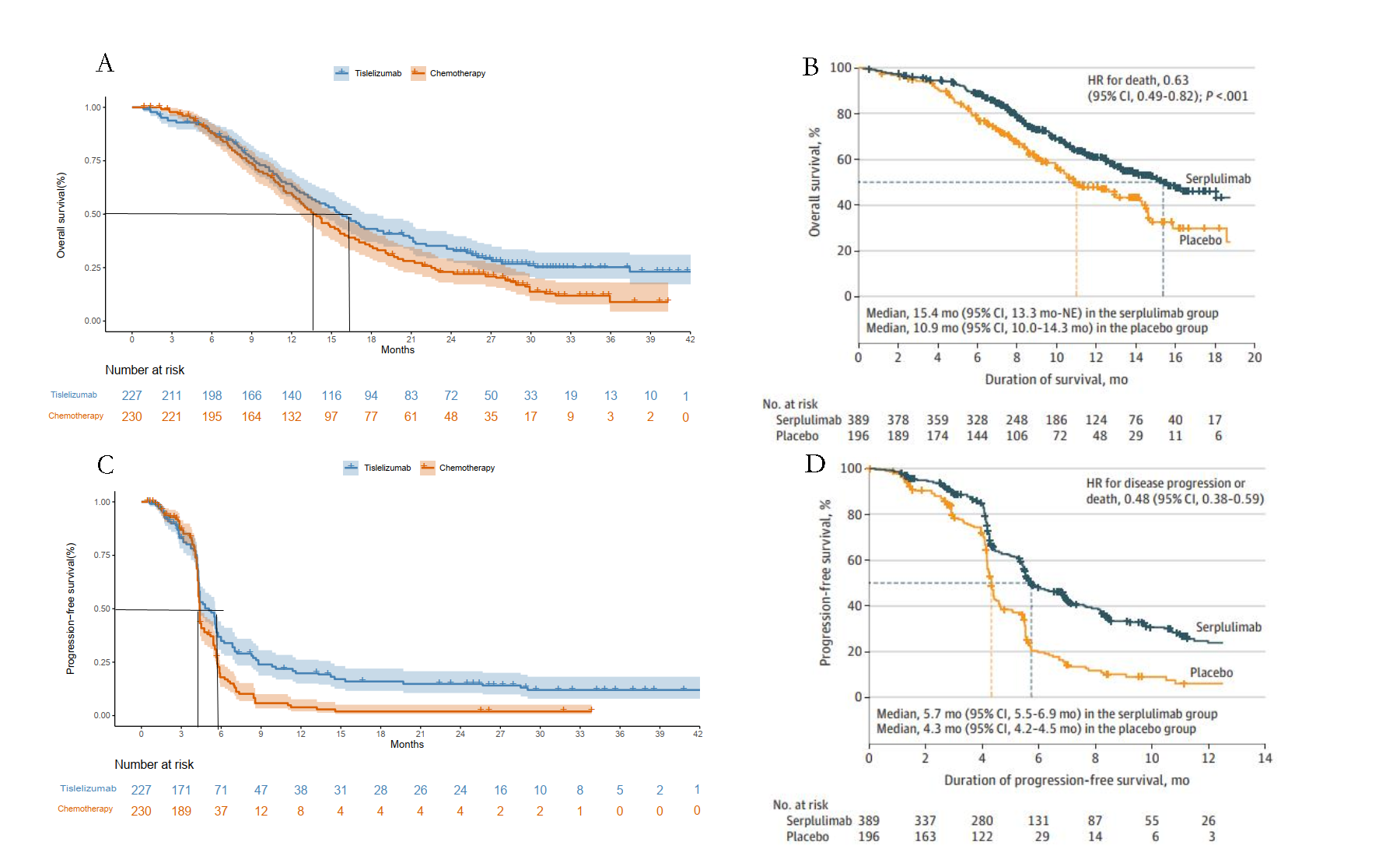

Supplement: S1 Fig — ;B: Overall survival curve from ASTRUM-005 clinical trial;C: Modes simulation visual progression-free survival curve;D:Progression-free survival curve from ASTRUM-005 clinical trial. (TIF) [file pone.0320189.s002.tif]

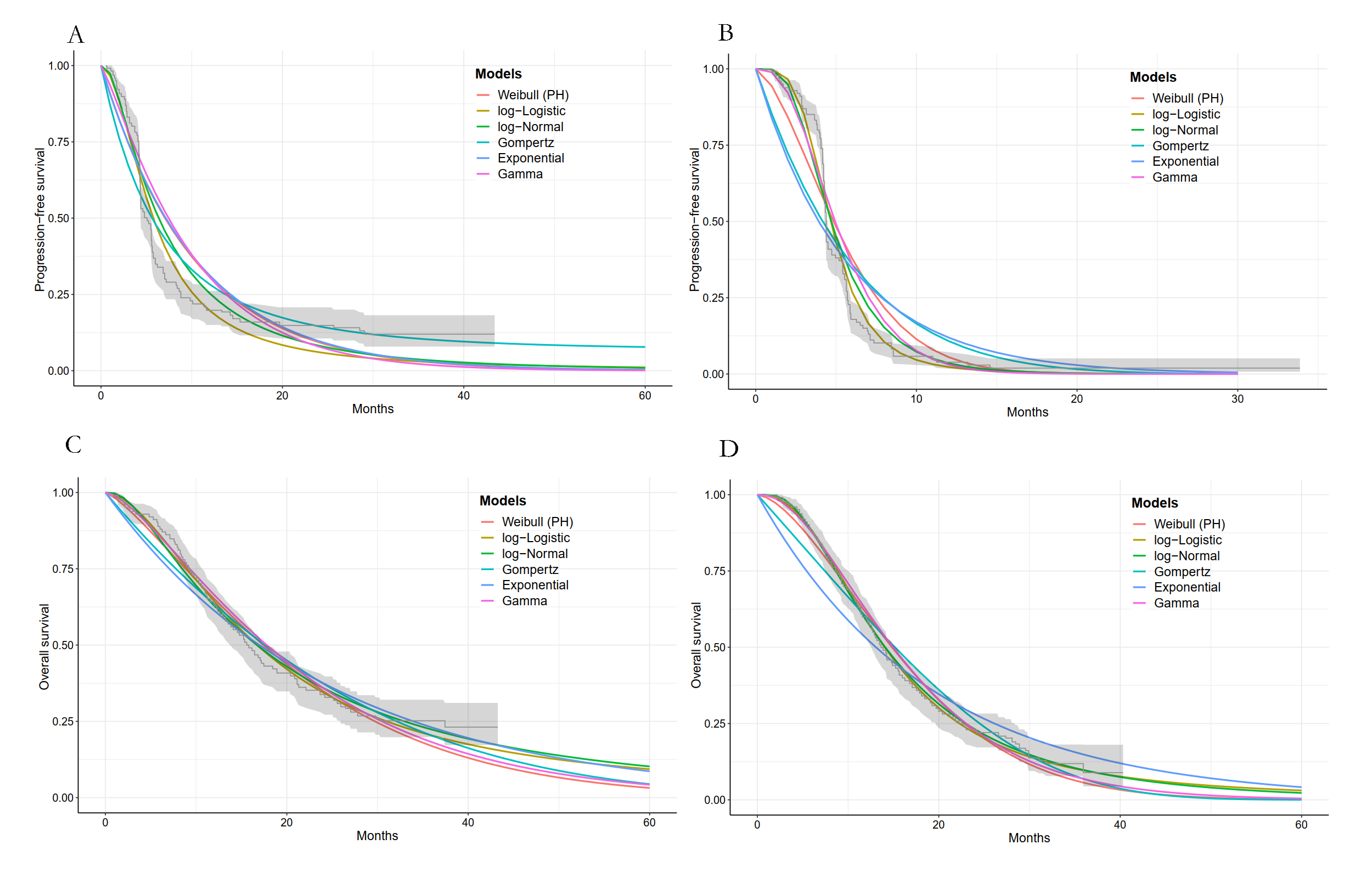

Supplement: S2 Fig — B:Modes simulation visual progression-free survival curve of chemotherapy group;C:Modes simulation visual overall survival curve of tislelizumab group;D: Modes simulation visual overall survival curve of chemotherapy group. (JPG) [file pone.0320189.s003.jpg]
